# Mucinous adenocarcinoma presenting as an isolated sternal metastasis

**DOI:** 10.1186/1477-7819-5-105

**Published:** 2007-09-24

**Authors:** Elizabeth Ball, Gareth Morris-Stiff, Mari Coxon, Michael H Lewis

**Affiliations:** 1 Department of Surgery, Royal Glamorgan Hospital, Ynysmaerdy, Llantrisant, Wales, UK; 2 1 Golygfa'r Eglwys, Maesycoed, Pontypridd, Rhondda Cynon Taf, CF37 1JL, Wales, UK

## Abstract

**Background:**

As a result of improvements in diagnostic accuracy, the primary source of the tumour is identified in more than 99% of cases presenting with a malignancy. Whilst the axial skeleton is a common site of metastases, the sternum is rarely affected, especially by isolated metastases.

**Case presentation:**

We report a case of a 68 year old male who was referred to the surgical outpatient clinic with a six month history of sternal pain. The patient was known to have essential thrombocythaemia, which had recently transformed into acute myeloid leukaemia but a sternal biospy showed mucinous adenocarcinoma. He had not localising symptoms and full evaluation failed to localise the primary tumour.

**Conclusion:**

Solitary sternal metastases are rare and when found an underlying neoplasm is usually identified allowing targeted treatment. If however, there is no symptomatic tumour, the metastasis should simply be treated symptomatically.

## Background

As a result of improvements in radiological imaging including cross-sectional and radiopharmaceutical studies, together with refinements in histological assessment of biopsy specimens such as the use of immunostaining, the primary site of a malignancy is usually identified and thus cancer of unknown origin now has an incidence of only 0.5–0.9% amongst all patients presenting with a malignancy [1].

Bone is the commonest site for secondary spread of cancer, with 80% of metastases arising from breast, lung and prostatic primary tumours [2]. The metastases are predominantly located in the axial skeleton within the spine, pelvis and ribs. Sternal metastases are rare; no series has been published recording the incidence.

## Case presentation

A 68 year old male was referred to the surgical outpatient clinic with a six month history of sternal pain. The pain was an intense, continuous burning pain that disrupted his sleep. The patient was known to have essential thrombocythaemia, which had recently transformed into acute myeloid leukaemia. This had been confirmed by a previous marrow biopsy taken from the iliac crest just before the onset of his sternal pain.

The patient had lost two stones in weight despite no loss of appetite. There had been no change in his bowel habit. He reported a single episode of rectal bleeding two months prior to his presentation that had been attributed to aspirin therapy. The patient admitted to smoking ten cigarettes a day but denied any respiratory symptoms.

On examination he was acutely tender in the mid-sternal region, but no mass was palpable. No breast or axillary masses were detected. Examination of the abdomen revealed three centimetres of hepatomegaly, a normal spleen but no other masses. Prostatic and rectal examinations were normal. Flexible sigmoidoscopy was also normal. A full blood count was consistent with his leukaemia. His renal function, liver function tests and prostate specific antigen tests were within normal limits.

**Figure 1:**
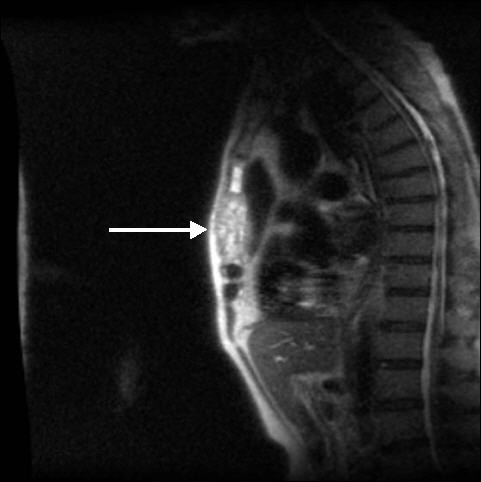
MRI scan demonstrating sternal metastasis with loss of marrow signal and abnormal cortical margins. The white arrow identifies the metastasis within the body of the sternum.

The patient underwent a magnetic resonance imaging (MRI) scan which showed abnormal marrow signal in the body of the sternum with expansion and loss of the anterior and posterior cortical margins (Figure 1). A computerised tomographic (CT) guided biopsy of the sternum was performed, histological examination of which identified a mucin-secreting adenocarcinoma. The patient subsequently had a full body CT scan and a bone scan. The CT scan was normal, specifically showing no abnormality in the thorax or alimentary tract. The bone scan confirmed a solitary metastatic deposit in the sternum. The patient was then referred to an oncologist for radiotherapy.

## Discussion

Cancer of an unknown primary site has a median survival of 9–12 months from initial detection [1]. The aim of clinical investigations in these cases is to obtain a biopsy of the metastatic lesion and to try to identify the source of the primary tumour.

Abeloff and colleagues [3] recommend the following series of tests: full history and examination, routine blood tests including tumour markers, chest radiograph and CT scan of the abdomen and pelvis. For those patients with no obvious primary tumour, the treatment will depend on the presenting metastasis. For a patient with a single bony metastasis, local treatment in the form of radiotherapy combined with analgesia is used to combat pain. If a patient has multiple metastases from an adenocarcinoma, some clinicians would consider giving systemic chemotherapy.

Bone is the commonest site for metastatic spread, especially in tumours involving the breast, lung, prostate, kidney and thyroid gland. The axial skeleton is usually affected, believed to be due to the vertebral-venous plexus, and the slow blood flow in the axial skeleton. Bony metastases present with pain and local tenderness, problems with mobility and pathological fractures. Although metastases to the vertebrae and ribs are common, sternal metastases are rare. Whilst there are several case reports of metastases from known primary tumours in the English language literature, there are only four series reported in the literature in the last sixty years [2,4-6]. However, these papers do not report an incidence as there is insufficient histological material to enable reliable conclusions to be made. Toussiet *et al*. [4] reported ten patients whose first presentation of a malignancy was a sternal deposit. Eight of the ten cases were haematological malignancies, and the other two were of renal and bronchial origin.

Mucinous adenocarcinoma such as described in this case may arise in the breast, lung, prostate, pancreas and bowel. Despite thorough investigation, we were unable to detect a source of the primary mucinous adenocarcinoma.

This case demonstrates that bony pain in patients with a haematological malignancy may be due to a second primary carcinoma. Sternal pain has many causes including neuropathic, musculo-skeletal and infective. Although metastasis is a rare cause of sternal pain, it should not be overlooked.

We question how aggressively a clinician should search for the primary site. With a life expectancy of less than a year, it may be kinder to the patient to restrict investigations to blood tests, a chest radiograph and a CT abdomen and pelvis. Further tests such as endoscopy, bone scans and barium enemas are uncomfortable for the patient and it may be argued that they should be avoided.

## Conclusion

Despite advances in medical imaging, there remain rare cases in which no primary can be identified for isolated metastases. In the case of bone metastases from adenocarcinoma, it may be appropriate to treat the metastasis symptomatically and not be too concerned with hunting the primary lesion.

## Competing interests

The author(s) declare that they have no competing interests.

## Authors' contributions

The original idea was that of MHL, the Consultant responsible for the case. The case report was written by EB and MC with assistance from GMS who edited the manuscript together with MHL. All authors approved the final version.
